# ILSI Health and Environmental Sciences Institute (HESI), global leader in advancing translational science to create science-based solutions for a sustainable, healthier world

**DOI:** 10.1186/s41021-015-0001-0

**Published:** 2015-06-16

**Authors:** Ayako Takei

**Affiliations:** ICaRuS Japan Limited, 3-4-2-4201 Toyosu, Koto-ku, Tokyo 135-0061 Japan

**Keywords:** Tripartite, NPO, Toxicology, Risk assessment

## Abstract

The Health and Environmental Sciences Institute (HESI) is a non-profit scientific research organization based in Washington, D.C., U.S.A. HESI was established in 1989 as a global branch of the International Life Sciences Institute (ILSI) to provide an international forum to advance the understanding of scientific issues related to human health, toxicology, risk assessment and the environment. For the last 25 years, HESI has been the global leader to advance application of new science and technologies in the areas of human health, toxicology, risk assessment and environment. The core principle of “tripartite approach” and the multi-sector operational model have successfully supported HESI’s scientific programs to create science-based solutions for a sustainable and healthier world. HESI’s achievements include the dataset to guide the selection of appropriate supporting assays for carcinogenicity testing, a new testing framework for agricultural chemicals with enhanced efficacy, predictivity, and reduced animal usage, novel biomarkers of nephrotoxicity which provide data on the location of timing of drug effects in the kidney allowing for enhanced drug development, etc.

## Introduction

The Health and Environmental Sciences Institute (HESI) is a non-profit scientific research organization based in Washington, D.C., U.S.A. For the last 25 years, HESI has been the global leader to advance application of new science and technologies in the areas of human health, toxicology, risk assessment and environment. The core principle of “tripartite approach” and the multi-sector operational model have successfully supported HESI’s scientific programs to create science-based solutions for a sustainable and healthier world. This article is an extended summary of “HESI Update” a presentation given by the author at the public symposium “Regulatory Science”, organized by the Japan Environmental Mutagen Society on May 24, 2014, Tokyo Japan. For the benefit of the readers, the contents were expanded to include some basic information on HESI, its organization, operation and achievements, and the latest activities.

### Tripartite approach and multi-sector operating model

The Health and Environmental Sciences Institute (HESI) is a non-profit scientific research organization based in Washington, D.C., U.S.A. HESI was established in 1989 as a global branch of the International Life Sciences Institute (ILSI) to provide an international forum to advance the understanding of scientific issues related to human health, toxicology, risk assessment and the environment. For over 25 years, HESI has successfully organized and supported a multitude of international scientific programs to identify and resolve global health and environmental issues. HESI’s programs bring together scientists from around the world from academia, government, and industry to address and reach consensus on scientific questions that have the potential to be resolved through creative application of intellectual and financial resources. This “tripartite (academia, government, and industry)” approach forms the core of every HESI scientific endeavor, and also the key to the success to create science-based solutions that can be accepted by both the private and public sectors. Currently, HESI’s activities involve around 90 universities and research institutes, hospitals and NGOs from 14 countries, over 50 government agencies from 12 countries, 60 multi-national sponsor companies from multi-sector private industries, and over 70 scientific projects executed via 15 different multi-sector committees. In order to fulfill its scientific mission, HESI adheres to a multi-sector operating model; the proposition that scientists from academia, government, foundations, health care, and industry should be fully integrated into all levels of the HESI’s activities, including governance bodies such as the HESI Board of Trustees, as well as in the work of scientific committees, task forces, and working groups. This principle is a fundamental aspect of HESI’s identity, and it is reflected in the daily operations of the programs. HESI enables successful teamwork among experts who bring their unique skills and viewpoints to the scientific and decision making process, and share responsibility for identifying research topics, designing and leading studies and projects, and interpreting and applying results via Scientific Committees.

### Governance, stewardship, and peer-review

HESI is governed by the Board of Trustees, comprised of 16 representatives from the public sector and 15 representatives of HESI’s sponsor companies, which provides scientific, strategic, and financial oversight for the organization, and by its Assembly, consisting of private sector sponsor companies. The HESI Assembly currently consists of 60 private companies from North America, Europe, Japan, Israel, and Singapore. The companies represent the chemical, agrochemical, pharmaceutical, biotechnology, cosmetic, consumer products, and food industries, and the CROs and the providers of materials for testing the products of these industries. Full lists of the Board of Trustees, and the private sector sponsor companies are available on HESI Website [1]. HESI operates according to the policies stipulated in its Bylaws [2], and with the principles of ethics and standards of conduct [3].

The HESI Stewardship Program, administered by the Program Strategy and Stewardship Committee (PSSC) of the HESI Board of Trustees, is a mechanism to ensure high quality, scientific content of HESI committee work products. The PSSC conducts periodic reviews of HESI committees based on the principles of empowerment, delegation, and trust. The overall purpose of committee reviews is defined in the guidance document [4] and to ensure the following:

- The committee under review has a clear set of objectives, a time plan to achieve the objectives, and a commitment to productivity.
- The approach or approaches adopted to achieve the committee’s objectives, as well as the committee’s work product(s), are based on the best and most credible science.
- The committee’s objectives are aligned with HESI’s overall goals and objectives.

To doubly assure credibility, transparency, and technical accuracy of any work product, HESI has been conducting an “internal” peer review before the submission of manuscripts and reports developed by its committees to scientific journals. The addition of the internal HESI peer review process, as a prelude to the typical journal review, is designed to ensure that organization’s work products achieve the excellence demanded by HESI’s multi-sector project participants. Peer review is institutionalized at HESI through specific requirements that are actively followed by all HESI committees. Once internal peer review requirements have been met, work products are submitted to scientific journals where they are subjected to the journals’ peer review processes. Traditionally, the Vice-Chair of PSSC served as the Editor-in-Chief of the internal HESI peer review process, however, the Board of Trustees recently approved the motion to remove this requirement to allow the Vice-Chair to focus on other duties and create opportunities for more extended Editor terms when feasible. In June 2014, Dr. James Klaunig, Indiana University School of Public Health assumed the position of the Editor-in-Chief of the internal HESI peer review process to work with HESI for 3-years term.

### Achievements with global impacts

As a non-profit, charitable organization, HESI’s mission is to generate information and scientific resources of benefit to global public health and environmental safety. In support of this objective, all HESI projects make a contribution to the scientific public domain via publication in the peer-reviewed literature, deposition of data in publicly accessible database, workshops and/or other public outreach efforts. The number of publications since HESI was established exceeds 260, and the high quality research results have been cited in the scientific publications of various fields. HESI’s work enriches the existing body of scientific evidence and improves our understanding of how to apply science to improve human and environmental health.

HESI committees generate impactful science via a variety of mechanisms, including designing and conducting novel laboratory research, pooling and analyzing existing data, crating decision frameworks and methodologies, and identifying scientific best practices. The following are selected examples of HESI scientific programs that have completed their multi-year work plans with measurable global impacts:

#### Alternative to carcinogenicity testing

The dataset [5] generated in this project continues to guide the selection of appropriate supporting assays for carcinogenicity testing. The program was designed in relation to a 1995 International Conference on Harmonization (ICH) Expert Working Group on Safety declaration that data from alternative assays could be used in safety evaluation in place of a second bioassay supplementing the rat carcinogenicity study. The $33 million collaborative effort, provided the first of its kind comparative data to guide the selection of either the p53^+/−^ heterozygous knockout mouse, the rasH2 transgenic mouse, the TgAC transgenic mouse, the homozygous XPA knockout and the XPA/p53 knockout, and/or neonatal mouse models as well as the Syrian Hamster Embryo transformation assays.

#### Agricultural Chemical Safety Assessment (ACSA)

A highly awarded and widely cited proposed testing framework for agricultural chemicals was developed through this project with an emphasis on enhanced efficiency, predictivity, and reduced animal usage [6–9]. The ACSA program recommendations are cited as the basis for the Organisation for Economic Co-operation and Development (OECD) Guideline for Testing of Chemicals-Extended One-Generation Reproductive Toxicity Study [10], and are referenced in the removal of the canine study requirement in the U.S. Environmental Protection Agency (EPA) pesticide testing guidelines. Additionally, the program is cited in two National Academy of Sciences (NAS) reports for impact and relevance, awarded the U.S. EPA Scientific and Technological Achievement Award (Honorable Mention), and the UK National Center for the Replacement, Refinement, and Reduction of Animals in Research “Highly Commended Prize.”

#### Biomarkers of nephrotoxicity

Novel safety biomarkers of nephrotoxicity were generated and established in this project, and the non-clinical data set on these urinary protein biomarkers gained in 2010 European Medicines Agency (EMA) and the U.S. Food and Drug Administration (FDA) qualification for use in toxicology studies [11–13]. These biomarkers have subsequently become routine in safety assessment throughout the pharmaceutical industry. The biomarkers provide data on the location and timing of drug effects in the kidney allowing for enhanced drug development.

#### Cardiac safety

In response to series of public health reports on drug-related cardiac arrhythmias (*Torsades des Pointes* - TdP), HESI undertook a program to evaluate the utility of nonclinical safety testing assays for predicting clinical TdP. The published results [14–17] of these studies served as supporting experimental data for the subsequent development of the S7B guideline by the ICH Expert Working Group – the first ICH guideline to use safety pharmacology data from a nonclinical safety setting to inform potential clinical risks. Since the implementation of this guideline, the occurrence of unanticipated TdP effects from approved drugs has been largely eliminated [18].

### Processes to initiate new projects

HESI’s initiatives are constantly growing and changing to address contemporary human and environmental health challenges. The Emerging Issues (EI) Proposal Solicitation process is HESI’s traditional and longest-standing project adoption process. This mechanism ensures a platform for broad input on new science, and creates an opportunity for all interested parties (public and private) to engage in project development without the hurdle of an initial financial commitment. Proposals are solicited once per year and anyone from the public or private sectors can submit a proposal. These proposals undergo detailed initial review by HESI’s multi-sector public/private Emerging Issues Committee (EIC). Two or more promising proposals are selected by the EIC for presentation at the HESI Annual Meeting. These priority proposals are then submitted to the HESI constituency for prioritization and voting. The most promising proposals will form the basis of new scientific initiatives within HESI, and receives funding from HESI for approximately one year. This funding includes a dedicated scientific program manager, expenses for meetings and teleconferences, and limited travel funds for public sector participants. Participants (both public and private sector) are not required to pay any fees during the first year. If the project moves beyond 1 year in duration, the participants (in conjunction with the HESI program manager) must develop a budget and identify committed funds to complete the proposed project. HESI will match those funds (up to an established limit) during the second year. Beyond year two, the project must be funded entirely by participant dollars.

In September 2014, the project on “Framework for Intelligent Non-Animal Alternative Methods for Safety Assessment*”* was approved by the EIC for HESI action based on its scientific value and impact. The project is expected to result in a set of consistent, internationally relevant criteria against which the reliability and fitness-for-purpose of new non-animal methods or approaches are assessed. The leaders of the new subcommittee are Prof. Alan Boobis of Imperial College London and Dr. J. Craig Rowlands of The Dow Chemical Company. The leaders work with a steering team composed of expert academic, government, and industry scientists to scope out the objectives, focus, and approach for the project and to initiate the activities under the EI Subcommittee in 2015.

In addition to the EI Proposal Solicitation process, HESI has the Resources-at-Initiation (RAI) process, which is a mechanism for responding to well-defined and time-sensitive projects. The RAI process includes requirements for dedicated funding up front by the project submitters, as well as tripartite engagement and relevance to the mission of HESI. The RAI proposals have to go through the three-stage review process, firstly by the senior HESI staff, secondly by the EIC, and finally by the Executive Committee of the HESI Board of Trustees. Proposals are reviewed as they are received, and a response (positive or negative) is provided to the submitter within 4–6 weeks of submission, which is much faster than the EI Proposal Solicitation process of about 1 year. The HESI Vaccines and Adjuvant Safety Project Committee is one example which was established through the RAI process and successfully organized a workshop in Amsterdam in October 2012, which brought together the collective knowledge of scientists from academia, industry and government to better understand the relationship between adjuvant and vaccine safety, with a focus on autoimmunity.

Also, the direct integration of a project into an existing HESI committee is appropriate for single party submitters (one scientist, organization, or company) whose idea is directly relevant to the mission and objectives of the targeted committee. The proposal should augment the current research portfolio of the committee. Cardiac Stem Cell Work Group established in the Cardiac Safety Technical Committee is one of the examples that a new proposal from a sponsor company was integrated into the existing HESI committee.

Details of the above processes to propose new projects to HESI are available on HESI Website [19].

### Expanding and enhanced partnerships

Over 25 years, the number of HESI’s partners in both public and private sectors increased dramatically, and a collaborative relationship has been enhanced between HESI and each partners, and particularly with governmental and international agencies.

The CIPA (Comprehensive *In Vitro* Proarrhythmia Assay) initiative which targets to revise the ICH S7a/b guidelines to evaluate proarrhythmia risks of drugs, is originated from a proposal by a FDA scientist that was adopted through the EI proposal solicitation process in 2012. After a workshop co-organized by FDA, Cardiac Safety Research Consortium (CSRC), and HESI in July 2013 [20], the CIPA initiative was started with expanded partners of FDA, EMA, Japan Pharmaceuticals and Medical Devices Agency (PMDA), Health Canada, Japan National Institute of Health Sciences (NIHS), CSRC, Safety Pharmacology Society (SPS), and HESI, with HESI staff providing project management and assistance.

The objective of the CIPA initiative is to facilitate the adoption of a new paradigm for assessment of clinical potential of TdP that is not measured exclusively by hERG block and QT prolongation. The new CIPA paradigm will be driven by a suite of mechanistically based *in vitro* assays coupled to *in silico* reconstructions of cellular cardiac electrophysiologic activity, with verification of completeness through comparison of predicted and observed responses in human-derived cardiac myocytes. It is envisioned that CIPA initiative will ultimately lead to modification or replacement of the existing ICH S7a/b guidelines and elimination of E14 guidelines. The details of CIPA initiative can be found at CIPA Website [21].

In September 2013, a Memorandum of Understanding (MOU) was signed between FDA and HESI regarding the partnership to promote drug development and safety assessment. FDA and HESI agreed to further strengthen their partnership to improve drug development and safety assessment through collaborative researches, training activities, interaction/cooperation of scientists from public and private sectors. The active participation of FDA’s scientists in HESI’s projects is expected to further expand with this MOU, which would promote discussions among tripartite scientists on the regulatory science issues affecting drug development and safety assessment. Also, MOU has made it possible for joint sponsorship of workshops and meetings between FDA and HESI, and thus, is expected to enhance their collaborations in the CIPA and other projects.

In November 2013, a collaborative agreement was finalized between the World Health Organization (WHO) and HESI to develop and maintain a freely accessible Internet database of training courses in chemical risk assessment. This database project is a part of WHO Chemical Risk Assessment Network, of which HESI is an official Network member. The purpose of the WHO Chemical Risk Assessment Network Training Database is to provide access to information regarding human health chemical risk assessment training courses. This database allows people to search for in-person and online undergraduate and post graduate programs, continuing education, and society-sponsored not-for-profit training courses related to human health chemical risk assessment, including those on general and advanced risk assessment methodologies, as well as supporting topic areas (e.g., toxicology, exposure science, statistics, etc.) Details are provided on the course title, location, dates, and cost, plus full contact details and links to the associated organization [22].

Another example of an enhanced relationship between HESI and international organizations is the support for OECD to improve testing guidelines for chemicals. In 2014, the Development of Methods for a Tiered Approach to Assess the Bioaccumulation of Chemicals Technical Committee provided technical input into an OECD Standard Project Submission Form (SPSF), a study proposal titled “*In Vitro* Fish Hepatic Metabolism.” The SPSF was submitted to the OECD by the US and the European Commission and was approved in April 2014. The SPSF provides an experimental context for the development of data to improve the modeled prediction of chemical accumulation in fish. The HESI committee will provide supporting experimental data for the SPSF via a multi-site experimental ring trial. Ring trial study design and materials preparation initiated in March 2014, and the experimental and analytical phase of the *in vitro* ring trial in support of the SPSF continues through 2015. This significant research program involves 6–10 different laboratories worldwide and participants from academia, government, and industry.

### Pillars of excellence and CITE initiative to advance translational science

In recent years HESI has put emphasis on the following three pillars of excellence:

- **Knowledge to Application:** Implementing fit-for-purpose scientific programs, engaging diverse stakeholders and disciplines.
- **Global Vision:** Engaging in and supporting global initiatives that recognize that science has no borders.
- **Future Leaders:** Providing training, awards, and mentorship to foster the skills needed to meet the challenges of modern safety sciences.

In 2012, HESI initiated CITE (Combining Interdisciplinary and Translational Expertise) to create a new global forum for senior expertise to help shape the future of the practice of translational and multi-disciplinary science. The CITE is comprised of a series of strategic outreach efforts, educational programs, and research collaborations overseen by HESI’s Board of Trustees, and aimed to develop collaborative principles that transcend HESI’s individual scientific committees. The CITE initiative helps build partnerships, methodologies, and frameworks that bridge diverse technical disciplines and sectors through development of innovative and efficient networks that advance science form discovery to application. These efforts are intended to bring values to HESI’s existing scientific committees as well as the applied science community at large.

In 2015, HESI’s Future Leader Travel Fund will be launched to provide awards for young scientists to support participation in publicly-advertised HESI-sponsored scientific meetings and training activities. The aim of this fund is to help build the next generation of effective and skilled translational scientists by providing direct access to the scientific content, and senior thought leaders present at these forums.

## Conclusion

For the last 25 years, HESI has been the global leader to advance application of new science and technologies in the areas of human health, toxicology, risk assessment and environment. The core principle of “tripartite approach” and the multi-sector operational model have successfully supported HESI’s scientific programs to create science-based solutions for a sustainable and healthier world. Advancements in science and technology, however, have created situations where there are many hurdles between basic research and application, and we need “multilingual scientists” who can communicate with researchers in different fields and disciplines [23]. Endeavour of HESI continues to support the future need of translational science to further contribute to global public health and environmental safety.
